# A Qualitative Assessment of Participation in a Rapid Scale-Up, Diagonally-Integrated MDG-Related Disease Prevention Campaign in Rural Kenya

**DOI:** 10.1371/journal.pone.0014551

**Published:** 2011-01-18

**Authors:** Timothy De Ver Dye, Rose Apondi, Eric Lugada

**Affiliations:** 1 State University of New York Upstate Medical University, Syracuse, New York, United States of America; 2 Uganda Virus Research Institute, Entebbe, Uganda; 3 CHF International, Nairobi, Kenya; Walter and Eliza Hall Institute of Medical Research, Australia

## Abstract

**Background:**

Many countries face severe scale-up barriers toward achievement of MDGs. We ascertained motivational and experiential dimensions of participation in a novel, rapid, “diagonal” Integrated Prevention Campaign (IPC) in rural Kenya that provided prevention goods and services to 47,000 people within one week, aimed at rapidly moving the region toward MDG achievement. Specifically, the IPC provided interventions and commodities targeting disease burden reduction in HIV/AIDS, malaria, and water-borne illness.

**Methods:**

Qualitative in-depth interviews (IDI) were conducted with 34 people (18 living with HIV/AIDS and 16 not HIV-infected) randomly selected from IPC attendees consenting to participate. Interviews were examined for themes and patterns to elucidate participant experience and motivation with IPC.

**Findings:**

Participants report being primarily motivated to attend IPC to learn of their HIV status (through voluntary counseling and testing), and with receipt of prevention commodities (bednets, water filters, and condoms) providing further incentive. Participants reported that they were satisfied with the IPC experience and offered suggestions to improve future campaigns.

**Interpretation:**

Learning their HIV status motivated participants along with the incentive of a wider set of commodities that were rapidly deployed through IPC in this challenging region. The critical role of wanting to know their HIV status combined with commodity incentives may offer a new model for rapid scaled-up of prevention strategies that are wider in scope in rural Africa.

## Introduction

The Millennium Development Goals (MDGs) reflect a concerted global effort, in part, toward improving health in developing countries. [1] Despite considerable progress toward the MDGs, unfortunately most countries, particularly within Africa, are not on a success trajectory that will lead to their achievement by 2015. [2] Lack of progress toward achieving the MDGs is largely attributable to limitations around scale-up of and access to effective intervention (i.e., increased utilization of existing interventions) rather than to a lack of cost-effective interventions. [3], [4] In addition to the logistical challenges inherent in scaling-up effective interventions, progress in overall health status is limited by overly “vertical” programs that promote uptake and programming only around a single disease [5], [6] and by difficulties with mobilizing populations rapidly on a large-scale. [7] While attractive in theory, more “horizontal” approaches to organization of health interventions that cut-across multiple diseases and levels of intervention are elusive in practice, though such approaches address the reality in many African communities of important risks and diseases that co-exist simultaneously. [8] Recently, the blending of a vertical (disease-specific) approach to expanding horizontal (systems) aspects impacting several related conditions has been proposed as common ground, [9] termed a “diagonal” approach [10] that could indeed impact health systems. [11]

With Africa as the focus of numerous large-scale investments through bilateral and multilateral global health programs, such as President's Emergency Plan for AIDS Relief, the Global Fund for Aids, Tuberculosis, and Malaria, and the Global Alliance for Vaccines and Immunization, for example, developing new models of rapid implementation and integration of effective interventions on a wide-scale is of crucial importance. [12], [13] The Integrated Prevention Campaign (IPC) in rural Kenya targeted rapid uptake on a large-scale to a bundled set of evidence-based interventions to help promote rapid progress toward achieving the MDGs, largely by simultaneously addressing HIV, malaria, and water-borne illness. The IPC package of interventions reflects rapid-scale-up, evidence-based approaches to population health improvement. For example, HIV testing remains remarkably low in many areas of the world and the World Health Organization supports rapid expansion and scale-up of counseling and testing to enable awareness of one's own HIV status to help prevent transmission and as an entre into care. [14] Further, Granich et al [15] demonstrated that universal testing combined with immediate treatment could substantially reduce if not eliminate HIV transmission in a high-burden area within a few years of near-universal coverage. Further, long-lasting insecticide-treated nets (LLINs) have proven an important malaria preventative [16] and their distribution and use forms a critical component of malaria control worldwide. [17] Finally, point-of-use water filtration offers low-resource regions in Africa rapid access to clean water for consumption, particularly in populations with high HIV-related disease burden. [18] While large population campaigns are common in public health, indeed forming an important historical foundation for public health intervention, [19] they typically target a single disease or specific intervention (e.g. an immunization). Recently, however, the concept of “care bundling” which combines several evidence-based interventions addressing multiple related diseases and conditions has shown promise in reducing infectious disease. [20] This qualitative evaluation assessed participant experience in a large, population-based care bundle addressing common and significant infectious disease in rural Kenya.

## Methods

### Ethics Statement

All participants provided informed, written consent. The Institutional Review Board of the Kenya Medical Research Institute (KEMRI), approved the study.

The IPC campaign occurred over one week's time in Lurambi Division of Kakamega District, Kenya, in September 2008. Approximately 47,000 people above age 15 in the district (of 51,178 estimated [21]) attended the campaign, which included the following components: HIV voluntary counseling and testing (VCT), distribution of a bundled package of interventions (condoms, long-lasting insecticidal bednets, water filters (LifeStraw® Family or Personal), and cotrimoxazole prophylaxis for those with HIV), and health education and advice. Men received a smaller packet that contained the LifeStraw Personal filter while women received the LifeStraw Family filter. The campaign was conceived by Vestergaard Frandsen and implemented by a team of local, national, and global public and private sector partners, including the Kenya Ministry of Health and the Kenya Medical Research Institute.

The approach to the campaign initiated with community mobilization and public health education activities, followed by mass campaigns in 30 sites. [21] Upon registration, participants received educational interventions and demonstration of commodities, followed by individual pre-test counseling. The 138 participants declining the test received counseling and the preventive care package. The majority of the participants (n = 47,173) consented to testing and received their results. Those participants testing HIV negative received post-test counseling and preventive messaging, and those participants testing HIV positive received post-test counseling, cotrimoxazole, referral to continued care and a follow-up plan.

The qualitative assessment consisted of in-depth interviews (IDI) and included individuals of known HIV status who attended the campaign, were counseled, tested and received their test results. Qualitative sampling targeted equal numbers of both HIV-infected and uninfected individuals, who consented to participate and had adequate locator information on file as having been obtained beforehand during the campaign. Inclusion criteria for the qualitative assessment included: known HIV status, aged 15 and above, attended the campaign, were counseled and tested for HIV, received their test results, consented at the time of the campaign to be contacted in their homes in the future for follow-up, and had adequate locator information available on file.

The in-depth qualitative interviews were conducted one-month post-campaign. Individuals participating in the campaign were informed about the study as they left the product distribution point and, if they consented to participate, were registered for inclusion and their locator information taken. In total, 34 interviews were conducted using systematic random sampling from the list of consenting participants, with a convenience target of including 20 HIV positive participants and 20 HIV negative respondents. Interviewing took place until redundancy was achieved, somewhat short of the original sampling targets (final sample included 18 HIV positive participants, 16 HIV negative participants).

At the start of the interview, participants were informed of the study purpose, reassured of confidentiality of information divulged and provided verbal consent to be interviewed. Since participants would be asked to disclose to the interviewer their HIV status as part of the interview, all interviewers underwent thorough training on asking sensitive questions. The interviewers also signed confidentiality agreements before the study commenced.

Using a semi-structured interview guide, participants were asked a series of open ended questions related to their experiences after participating in the week-long campaign, which included VCT, product distribution and training, and education and counseling. Participants were asked about their satisfaction and experiences with the campaign, and also were asked to make recommendations for future improvements.

The qualitative interviews were audio-taped, transcribed and translated from Kiswahili and local languages into English. Text sections were coded by a pair of experienced social scientists and analyzed using NVivo 8 (QSR International, Cambridge, MA). Code lists were developed both from study objectives and from the data itself. Specific codes related to the topic were then selected and text examined for recurring themes and to find illustrative quotes to elaborate on specific findings from the analysis. [22]

## Results

As shown in Table 1, the majority of interviews were conducted in Kiswahili and lasted an average of about an hour and twenty minutes. Participants were a mean age of 38 years old, with a median of five children. Most participants had attended some form of secondary school, and most were farmers. About half of all participants were HIV positive, and about half of all participants were women.

**Table 1:** Interview and participant characteristics: qualitative assessment of the integrated public health prevention campaign against HIV, malaria, and diarrheal disease, Lurambi, Kenya.

Characteristic	Total (N = 34)
**Interview Characteristics**	
Length of Interview, median in minutes (IQR; interquartile range)	77 (59–139)
Language of Interview (n, %)	
Kiswahili	28 (82%)
English	3 (9%)
Local language	3 (9%)
**Participant Characteristics**	
Gender (n, %)	
Female	16 (47%)
Male	18 (53%)
Age, median in years (IQR)	41 (29–53)
Children, median (IQR)	5 (2–7)
Occupation (n, %)	
Farmer	22 (65%)
Other	12 (35%)
Education (n, %)	
Primary School only	10 (29%)
Some Secondary School	24 (71%)
HIV Status (n, %)	
Positive	18 (53%)

### I. Motivation for participation in IPC campaign

**“I want to know my status”.** The consistent single motivation for participation in the integrated campaign reported by assessment participants was the desire to know their HIV status (n = 31), coupled with the availability of the free HIV test (n = 31). The strength of wanting to know their HIV status and the appeal of the free HIV test was equally strong among HIV-positive and –negative participants.

“What motivated me was the idea of a free test for HIV. I said 'I have to go and get tested' so I can know my status.” (HIV-negative man, mid-40s)

“What made me go to the campaign was the need to know the status of my body. I just wanted to know the health status of my body.” (HIV-negative woman, mid-30s)

“I went to know my status. It made me go there.” (HIV-positive woman, early-30s)

Participants frequently mentioned already having known their HIV status previously, but were interested in the free test to learn if their status had changed (or to confirm their original result).

**“I got good services, and a good package”.** In different ways, all 34 participants in the qualitative assessment indicated that the availability of free goods and services also motivated their participation. Namely, as shown in Table 2, the mosquito net and water filter/straws were most commonly mentioned after the free HIV test (and among HIV-positive participants, the availability of free HIV-related medications).

**Table 2:** Characteristics motivating participation and satisfaction: qualitative assessment of the integrated public health prevention campaign against HIV, malaria, and diarrheal disease, Lurambi, Kenya.

Characteristic	Total (N = 34)
Mentioned wanting to know HIV status	31 (91%)
Mentioned free goods/services	34 (100%)
Specific item mentioned:	
Bag	12 (35%)
Mosquito net	28 (82%)
Water Straw/Filter	24 (71%)
HIV-related Medications (Offered n = 18)	16 (47%)
HIV Test	31 (91%)
Condoms	14 (41%)
Mentioned knowledge/advice	23 (68%)
Mentioned community mobilization	4 (12%)
Indicated satisfied campaign	34 (100%)
Indicated non-campaign-related unmet need	10 (29%)
Indicated suggested for future improvement	16 (47%)

“We were not able to purchase good bed nets. They gave us them for free, and we feel good when we are sleeping in them.” (woman, late-40s)

“Before they used to sell bed nets. I never thought that I could buy one. When they brought the bed net I can sleep in it and the mosquitoes and other insects don't disturb me. Before the net, you could be bitten with night insects and be forced to go.” (woman, early -30s)

“On the side of malaria, I was given a net to use, and to prevent me from diarrhea I got the water filter.” (woman, mid-40s)

While men and women relatively equally noted the appeal of free nets and water filters/straws, some gender difference was noted in mentioning the availability of other free goods (data not shown). Specifically, men were more likely than women to note the appeal and importance of free condoms (56 percent and 25 percent, respectively), while women were more likely to note the appeal and importance of the free bag (56 percent and 17 percent, respectively).

In general, the availability of free goods and services appealed to IPC campaign participants.

“He [the village chief] said a care package would be given, and that we would be given nets and water filters free-of-charge. It turned out to be true! I discovered that I was given good things.” (man, mid-30s)

Overall, the combination of providing HIV testing and free, useful commodities to prevent other non-HIV-related diseases formed a strong incentive for participation.

“What I liked was the net and the water filter. And also I liked being tested for HIV. What I liked most was knowing my HIV status. That is what I was looking for.” (woman, early-50s)

**“I went to see what it was all about”.** Finally, a few participants mentioned that the IPC campaign was a community event, either endorsed socially or through village leadership, and that they attended the campaign because it was an appealing social activity. Participants note that the campaign was endorsed by local leaders, or otherwise provided an interesting community event.

“If there is something new that comes up, it is good to find out what it is, so I went to see what it was all about.” (woman, late-40s)

“I was motivated to attend the campaign because when I heard the announcement and posters, I thought it was necessary.” (man, early-20s)

“The village elder had said in the village meeting that no one should fail to go to the campaign.” (woman, late-50s)

### II. Experiences During IPC Participation

**“It is better to get tested…”.** The mass campaign was generally perceived to have provided to all people in the community a chance to know their HIV status, and promoting good health among communities. Participants reported that in some neighborhoods, people have come to understand and appreciate that those community members with a diagnosis of HIV/AIDS are not necessarily dying, nor that the person should be ashamed. This attitude has led to more open discussions about HIV testing and willingness to attend other HIV-related promotions.

“What I know is that long time ago people had known this disease as ‘**Eshikhura**’ [a disease that comes in a family and leaves makes somebody emaciated before dying]. I have heard stories that such patients were well taken care of so that he or she does not suffer from stigma. The family used to help the patient, for instance, there were traditional medicine for that. Sometimes people used to say the disease was a kind of a curse in a family if one had made a mistake. People treated such patients differently. There are those who had mercy on such patients while others acted differently”.

(HIV-infected man in his 50s)

“……nowadays people know that if we direct a person well until they get drugs, they will live until they reach a point of dying, if we love and help them they will feel happy. There was a time an AIDS campaign was held in Kakamega, we went to sing there, then men and women who are in the club for PHAs [people living with HIV/AIDS], I was even telling people that I live with the virus, people could not believe but the campaign has made people more strong, even some sat that there is no problem, for a human being you cannot escape it and if you have it, it is better to get tested that you can know how you will continue with your life” (Woman not infected with HIV, in her 50s)

**“They are now seeing that there is hope”.** Most participants indicated that there was general change in the acceptance of HIV-infected persons, unlike they experienced in the past. One HIV-infected man said people were afraid to go out for an HIV-test on their own because it meant they were aware of their status. The campaign was understood to have made HIV a situation that anyone could have and therefore increased the acceptance of the disease and HIV-infected persons. The mass campaign was labeled as a tool to reduce stigma especially because the whole community was given equal opportunity. It was mentioned though, that women were more prone to attend such campaigns than men. The acceptance of HIV in the community is illustrated by a quote from an HIV-infected participant;

“Yes, there is change, right now people thought that the lives of people with HIV patients was very short, but once they personally took the initiative to do the test, they discovered that even when one tests positive there is hope because you can start going to the clinic and life continues being normal, they are now seeing that there is hope in the lives of HIV infected people. They are being taken by others in the village as part of them and they offer support and encourage them, they now feel they are part of the society”. (HIV-infected man, in his 40s)

**“They gave very good advice”.** In addition, another dimension of the IPC campaign that participants found motivating was the availability of counseling, knowledge, and advice: about two-thirds of participants mentioned that they appreciated the lifestyle advice provided by campaign workers (see Table 2).

“They have a lot of advice on how people can live and avoid getting illnesses that are dangerous to your life.” (man, early-40s)

“I would attend [a similar campaign in the future] because they gave me useful information.” (man, late-20s)

“Even my husband likes it very much, because campaigns usually have good advice.” (woman, mid-20s)

### III. Post IPC Participation Experiences

One female participant's partner did not want her to go for HIV testing or ART eligibility evaluation, reported that whereas she needed to seek treatment, starting with an HIV test, to save her life; this was a course of action to which her husband was opposed citing that this would amount to stigma from others. This participant had to disobey her husband to save her life, causing disruption to the relationship. Another female participant reported that being singled out for the qualitative interview maybe associated with her sero-status presenting a possible danger of public disclosure of her HIV sero-status. Occassionally, the public was construed to making interpretations of one's sero-status using the basis on what one received from the campaign.

“Even when I came back here from the campaign, people thought that the different color of the bags had different meanings, they were saying that the people with red bags have AIDS but those with blue and black ones don't have AIDS. I was given the blue one so they just don't know how wrong they were They treat this people like that because they think that these people are the kind who are not faithful to their partners so they are not trustworthy people.” (HIV-infected man, in his 40s)

There were mixed opinions about how HIV-infected individuals should be treated. This is illustrated by the quotes below;

“They should forget about marriage and stay like that, they should take care of their children.” (HIV-positive woman, in her 20s)

“People living with HIV have a right to employment, it is okay for them to work but when they start getting sick they have to stop working and be given assistance to sustain their lives.” (HIV-negative woman, in her 30s)

### IV. Satisfaction, suggestions for improvement, and other unmet community needs

**“The campaign was brought at the right time”.** All 34 assessment participants indicated they were quite satisfied with at least some dimensions of the IPC campaign (Table 2). Most indicated that they were satisfied with the content of the campaign, that is, the free goods, testing, services, and health education.

“I have benefited from what they gave me because they are useful to me. They help me because I no longer drink dirty water, I also have a bag to carry my things inside. The net prevents me and my children from being bitten by mosquitoes.” (woman, late-40s)

“I did not see anything bad. Those health officials did a good job. The campaign was brought at a good time.” (man, early-50s)

“All that they did was good. That I know my health status as I live, that I have to live until my last day on earth.” (woman, mid-40s)

Some participants specifically noted that the style and approach of the campaign staff to organize and reach participants.

“They were talking and teaching well.” (woman, early-30s)

“They made a lot of fun. People were amused! They really made us happy, they were not tiresome.” (man, early-40s)

“They had organized the place so well, there were good tents, chairs, water tanks.” (man, late-50s)

Overall, participants viewed the campaign as well-run, and successful in that the services they received were worth the efforts to obtain them.

**“People were so many!”.** About half of the assessment participants offered criticisms or suggestions for improvements in future campaigns. Men were more likely to offer suggestions for future campaign improvements than were women (61 percent and 31% respectively), though offering criticisms or suggesting improvements did not vary with HIV status. What criticisms and suggestions were offered centered around three themes: 1) perceptions of favoritism in the queue process, 2) logistical challenges, and 3) not receiving goods expected.

Regarding perceptions of favoritism, some participants noted that they thought some people were moved ahead in the queues for unclear reasons.

“The only people I did not like were [mentions a different tribe] because they were discriminating. They wanted people from their villages to be served first.” (man, early-50s)

“They should not have elders attending to the queue. New people should come so those that don't know us can attend to us, so that there are no bribes.” (man, early-30s)

“It was the issue of favoritism that I was not happy about.” (woman, late-50s)

Additionally, perhaps as expected with the size and nature of this event, some suggestions centered around queue management and waiting.

“First of all, people were so many in the queues and the people there were making so much noise.” (woman, early-40s)

“People were many, they squeezed on the queue and that made me stay longer.” (woman, early-40s)

Finally, some participants did not receive the goods they had expected, either due to the distribution protocols, or because supplies ran out.

“What I can say about the family package was that the size was small. I wish they brought large sizes when the campaign is brought again. This can make work easy for my wife.” (man, early-50s)

“The family water filter, they should not have said that it should only be given to females. I was not happy because presently I am staying alone. [When I have visitors], I have to find other means of getting water.” (man, early-50s)

“I would like to see them bring in more bed nets and bags and also water filters if they can.” (man, early-40s).

**“I would like health centres brought close to the people”.** Overall, ten participants mentioned other unmet needs in the community, which could either enhance the effectiveness of future campaigns, or which could interfere with the potential effectiveness of campaigns. Men were more likely to note other unmet community needs than were women, and HIV-positive participants were similarly more likely to mention other unmet community needs than were HIV-negative participants.

Most comments grouped in this theme addressed deficits in the existing health system in the region, especially with access to care in a remote and impoverished area. Several participants noted that expanding permanent health services would improve the ability of campaigns such as the IPC campaign to be effective.

“If this campaign were to be repeated, I would like health centers brought close to people. Some people come from far to attend clinics in these centers.” (HIV-positive man, early-50s)

“Truly speaking, they should organize and bring a hospital around here. We are very far.” (HIV-negative man, early-30s)

Other comments around unmet needs surrounded other issues of supplies and information beyond the scope of the IPC campaign, and what the campaign was designed to provide.

“They (sick people) can be brought things like clothes, or money for use, because you have to eat well.” (HIV-positive woman, early-20s)

“What I would like to be done differently is give support to people living with HIV/AIDS food, and even transport when going for medicine.” (HIV-positive woman, late-40s)

“I was thinking if they come back they could make us benefit with things like money, or things like blankets or mattresses. Others do not have blankets in their house.” (HIV-positive man, early-30s)

## Discussion

The recent increase in funding for healthcare in Africa merits innovation in delivery of health services to maximize population benefits. [23], [24] The Integrated Prevention Campaign (IPC) used a multi-disease health campaign targeting Human Immunodeficiency Virus (HIV), malaria and diarrheal diseases to maximize health outcomes to the population. Population-targeted mass campaigns have, previously, successfully been implemented for single diseases such as malaria and HIV voluntary counseling and testing (VCT) interventions. [25], [26], [27]

In this study, delivery of health interventions using the mass campaign model of delivery of care and services including HIV testing for the public did not result in a high prevalence of negative social experiences perceived to be related to study participation. The prevalence of negative social experiences related to study participation was low and is further illustrated by the minimal reports of first-person negative social outcomes in the qualitative study.

This study reflects a qualitative assessment of participation in a mass public health campaign, aimed at rapid community mobilization, education, and distribution of effective interventions to reduce morbidity and mortality from a range of preventable causes. The aim of this assessment is not to provide numeric ascertainment of correlates of campaign participation but rather to identify themes and patterns that can perhaps inform future campaign design and implementation.

Themes regarding motivation for participation in IPC clearly emerge from this assessment: campaign participants desire to know their HIV status. Secondary to knowing that status, and participants desire access to interventions that help prevent very real health problems facing their communities and families. Participants desire the opportunity to learn about preventing health problems and to discuss their own health with professionals. Finally, some participants are motivated to the campaign because it's an event, something interesting to do, or because their village leaders encouraged their attendance.

The voluntary counseling and testing (VCT) component of this campaign was a strong motivator for community and individual participation, and served as an incentive for attendance and as a catalyst for access to the range of interventions provided. That VCT was bundled with other health interventions in an effective manner to mobilize a large population rapidly is an encouraging demonstration of “diagonality,” integrating access to one service (VCT) to stimulate access to other evidence-based interventions (mosquito nets, water filters, condoms) that, when taken together, address the top burdens of disease in East Africa. Often drawn to the campaign by the opportunity for VCT, community members appreciated and welcome the potential to address other diseases such as malaria and diarrhea, and responded very well both to the goods distributed and to the education provided.

Debate surrounds innovation in provision of HIV care if models present risk of publicly identifying clients as people infected with HIV. Thus, to the extent that HIV is a highly stigmatized condition, public disclosure that occurs as a result of health care provision could prove dangerous to persons receiving such care. [28] Some studies have documented HIV disclosure being associated with positive social outcomes for participants. Other studies found, however, that non-disclosure continues to be an obstacle to care and prevention. [29] This obstacle seems to be particularly the case when participants say that their non-disclosure is linked to fear of stigma. [30], [31] Some studies have shown that HIV positive individuals avoid sharing test results with family, friends and most importantly sexual partners. Barriers to disclosure include fear of abandonment, loss of economic power, discrimination, violence, upsetting family members, and blame. [32] For all of these reasons, any attempt to realize the possible gains that could be made by a mass campaign that includes VCT should only be attempted with attention to avoiding unintended negative social outcome for program participants.

While the qualitative data demonstrate the prevalence of negative and positive experiences perceived to be related to participation in the mass health campaign program, the data allow us to understand the contextual nuances and the stories associated with these experiences. We understand from these data that the HIV testing and counseling component of the program was valued by participants, and that positive experiences and avoidance of negative experiences were often attributed to the provision of HIV testing and counseling. This supportive counseling was invaluable with regard to addressing issues related to referral to care and HIV prevention risk reduction, and attributions of acceptance of HIV within the community that would lead to less stigmatization of the disease.

Satisfaction was clearly enunciated by campaign attendees participating in this assessment. People appreciated the opportunity to receive real benefit for diseases they experienced as important in their communities, they enjoyed the manner in which the campaign was implemented, and they felt positive about their participation. Maintaining a high level of satisfaction with a campaign such as this one may encourage positive health behavior change in attendees and could well support future participation in similar campaigns. [33]

The large-scale mobilization of populations for such campaigns that combine access to medical service and care, distribution of free, valuable goods, and the presentation of health education, however, are not without some (perhaps expected) logistical limitations. Perceptions of favoritism or differential (or otherwise inequitable) access to the benefits of the campaign are complicated by long-established social distinctions that are often embedded in communities. Perceptions of discrimination by age, gender, or ethnicity commonly complicate provision of services, whether manifested authentically in the provision of such services or whether understood by populations to be the case. Clear understanding of community dynamics prior to deployment of a large-scale campaign and efforts to reduce misperception of favoritism would help minimize the potential disincentives that such perceptions could have on participation.

Finally, additional considerations for integrated campaigns that could limit effectiveness are other, non-campaign-related unmet needs. Participants describe concerns about local health systems' inabilities to support on-going continuous care and resources for complex diseases such as HIV, and that other tangible needs such as food, clothing, and shelter could limit the effectiveness of the interventions deployed in the campaign. Designing and implementing a large-scale integrated campaign in the context of pre- and post-existing health and social systems provides an important component of sustainability and continuity that may enhance the effectiveness of the campaign.

Several limitations qualify this study. First, the qualitative data represent themes and constructs evident in the sample and are suggestive of themes found in the larger participant population. The sample, however, is not necessarily representative of all IPC attendees and the data cannot be numerically generalized. Further, the qualitative assessment did not include the participants refusing HIV testing altogether (which accounted for 0.3% of the overall IPC attendee population); these qualitative data therefore cannot assume to be representative of that population.

This qualitative assessment showed that motivation for participation and satisfaction with an integrated campaigned designed to rapidly deploy evidence-based intervention are strong and meaningful. As a qualitative study, the assessment cannot quantify various dimensions of participation and satisfaction for generalization to the wider scope of campaign participation, but it can provide useful information to help continually improve and streamline the design and implementation of similar campaigns in the future. Given the large volume of participants engaged with the campaign and its short duration, coupled with community perceptions of usefulness and satisfaction with their own participation, this type of integrated campaign may well become a new model for engaging populations and, possibly, for reducing morbidity and mortality associated with common and preventable risks.
